# Strategic yet delicate: the dilemma of involving health workers in facilitating birth registration in Indonesia

**DOI:** 10.1186/s12913-019-4594-z

**Published:** 2019-11-26

**Authors:** Clara Siagian, Wenny Wandasari, Feri Sahputra, Santi Kusumaningrum

**Affiliations:** Center on Child Protection and Wellbeing (PUSKAPA), University of Indonesia, Depok, Indonesia

**Keywords:** Birth registration, Civil registration, Vital statistics, Health workers, Healthcare, Health services, Indonesia

## Abstract

**Background:**

Birth registration provides the basis for population data. Previous studies have examined that collaboration between the health sector and civil registration can help improve birth registration rate. However, there was a little exploration into health workers’ understanding of civil registration and vital statistics (CRVS) and their perceived role in it. This study aims to fill this gap by focusing on the perspective of both health personnel in a managerial position and those who are involved in direct service provision to the community. Finally, we discussed the opportunities and challenges to strengthen the birth registration presented by health workers’ diverse views.

**Method:**

This study uses a qualitative approach through semi-structured in-depth interviews with 23 provincial to village health personnel in Pangkajene Kepulauan (Pangkep) district of South Sulawesi province. The participants were selected through consultation with the Department of Planning and the head of the Department of Health at provincial and district level based on the relevance of their position with CRVS. At the frontline level, the informants were identified using a snowballing technique and recommendation from community members.

**Results:**

This study finds that at the village level, health workers perceive CRVS as important since it supports them in delivering healthcare to community members. They see identification document like birth certificate as crucial for healthcare seekers to access the government’s health insurance and with that, proper and affordable treatment. Some health workers have been facilitating birth registration on a discretionary basis. Local health officials agree that accurate birth data lead to effective planning and financing for healthcare services and insurance. Despite the positive perception of birth registration, the majority of health workers do not want the additional burden for registering births. Health officials, however, are more open to taking some responsibilities.

**Conclusion:**

This study concludes that the level of health workers’ understanding and appreciation of the CRVS system provides opportunities to engage them systematically in birth registration. It recommends that institutionalizing health workers’ participation in birth registration must consider their current workload, revision of legal instruments, capacity building plan, and operable linkage with civil registration authority.

## Background

In 2015, the government of Indonesia pledged to increase the ownership of birth certificates for children below the age of 18. It aims to raise the coverage to 85% by 2019 for all children and to 77.4% for children from poorer families [1]. In addition to being a national priority, Indonesia commits to an increased birth certificate coverage through the Asia Pacific CRVS Decade 2015–2024 [2] and the Sustainable Development Goals (SDG) specifically target 16.9 “to provide legal Identity for all, including birth certificates, by 2030” [3].

In the last five years, Indonesia has made several breakthrough policies and has moved toward the digitalization of registration of vital events [4, 5]. However, it still has the second lowest coverage of birth registration in the region, after East Timor (Kusumaningrum et al: A child’s growth is a nation's growth: children’s wellbeing and inequlity in Indonesia, forthcoming). As of 2016, our analysis of the national socioeconomic survey (Susenas) found that 18% of children do not own a birth certificate (Central Statistics Agency: National socioeconomic survey 2016, unpublished). According to a study conducted in 2014, 73% of those who claimed to have a birth certificate but could not show it, actually mistaken birth certificate with other documents [6]. Therefore, if we include those who claimed to have a birth certificate but could not show it to the enumerator, the figure increases to 33%. With a total of 84,6 million children estimated by Susenas 2016, this leads to 28 million Indonesian children without a birth certificate.

Birth registration is one of the elements in civil registration and vital statistics (CRVS) system [7]. CRVS is a two-pronged mechanism that confers a state’s recognition of vital events to individuals through the provision of legal identity documents and produces data on the features of these events [8, 9]. The scope of vital events depends on a country’s legal framework, but it usually includes events of birth, death, the cause of death, marriage and divorce, and adoption [10]. Birth certificate facilitates a child’s access to the state’s protection and services including health care, social welfare, and education [11]. It is also considered as “the breeder document” that facilitates ownership to other legal documents such as passport and ID card [12]. Birth registration is also considered part of the vital statistics that produce timely and accurate primary population data [7, 13, 14]. Without an accurate number of population, births, deaths, and the causes of these deaths at any given period, governments including health sector cannot monitor indicators and evaluate interventions.

Countries with well-functioning CRVS have been found to have better health indicators than countries with a rudimentary system [15]. WHO asserts that the health sector does not only benefit from functioning CRVS systems, but it should play an active role especially in the recording of vital events [16]. In countries, such as Bahrain, Qatar [17] and Maldives [18], health workers are mandated to register and certify birth and death while in Thailand and Chile, health workers input details of birth and death into a shared database for civil registrars to issue certifying documents [19]. Not only this strategy increase the coverage of registration, but also cut the time lag between the occurrence and the registration of the event [20, 21]. Some studies attributed the increasing coverage of birth registration to the involvement of health workers [21–23].

Such streamlined practices between health and civil registration do not exist in many countries, including in Indonesia. Considering the low birth registration rate, Indonesia is a textbook case where the involvement of the health sector can bring significant improvement to the coverage, thereby increasing the accuracy of data produced. However, despite considerable efforts to increase birth registration rate in Indonesia, the involvement of the health sector has not been instigated [24, 25].

Appraisals on how the health sector can contribute to improving birth registration in several different countries are available [16, 19, 21, 23], including the strengths and weaknesses of such an approach. However, very little discussed the health workers’ standpoint of their role in birth registration. Integrating birth registration into the health system depends in part on the motivation of the frontline health workers and managers, therefore understanding their perspectives is critical to informing the current or future birth and other vital registration strengthening programs. This study investigates how health workers and managers identify their role in CRVS, especially birth registration, their perceived value of the system, and their observations on how they can and should play a role to make birth registration more effective.

## The policy context of civil registration and vital statistics in Indonesia

### CRVS in Indonesia

The United Nations defined civil registration as “the universal, continuous, permanent, and compulsory recording of vital events provided through a decree or regulation in accordance with the legal requirement of each country” [14]. In Indonesia, civil registration is part of a more extensive system called population administration that is codified by two national laws and several implementing regulations. The Law No. 23 of 2006 serves as the overarching regulation partially amended through the Law No. 24 of 2013. The amendment introduced several reforms, including the removal of court approval for late birth registration of over a year. The Ministry of Home Affairs (MoHA) is the leading authority of civil registration in Indonesia. At the subnational level, the Office of Population and Civil Registration (Disdukcapil) at the district level under MoHA is responsible for providing the services for population registry, to record all vital events, and to issue the appropriate documents. Some functions can be delegated to lower level of government, either to the sub-district that administers several villages or directly to the villages, but only a few districts implement this approach.

Every newborn needs to be registered to Disdukcapil no later than 60 days after birth. The application requires: (i) birth notification letter from a birth attendant, (ii) IDs of both parents, (iii) parents’ family card (KK) and, (iv) valid marriage certificate of the parents. If application is successful, Disdukcapil will issue birth certificate and add the newborn into the family card. Since 2010, each will receive a unique identity number (NIK) that appears on the birth certificate, KK, and IDs. Although the government does not apply administrative fee, applicants usually pay for transportation costs to go to Disdukcapil at district capital, and the process can take more than one visit.

There is no penalty for parents who don’t register their child, but the documents are required to access various services such as healthcare and education. To register to national healthcare insurance (JKN), individual need an NIK that appears on the birth certificate and KK. Although it is not a mandatory requirement for school enrollment, most schools request birth certificate during enrollment and/or national examination to register students into national education database.

Against this background, the government still struggles to ensure the universal ownership of birth certificate. Analysis of Susenas 2016 shows that almost 34% cited their inability to bear the associated cost as the reason for not possessing a birth certificate (Central Statistics Agency: National socioeconomic survey 2016, unpublished). The second reason was their birth certificates had not been issued (20%). Around 12% did not see the value of registering births while more than 9% did not know the procedures or that birth must be registered. Approximately, 7% of these respondents cited the distance to registration office as the barrier to having a birth certificate.

Due to the gap in registration, including birth, vital statistics informed by MoHA data has not been reliable. Other ministries run their own reporting channel and databases on vital events for programmatic purposes [25]. Although parallel yet interconnected information systems exist in most countries [26], in Indonesia these databases are not interoperable. Superimposition, exchange, and comparison of records rarely happen although an effort to develop interconnectivity is slowly underway.

In 2010, MoHA launched a population administration management system called Sistem Informasi Administrasi Kependudukan or SIAK. Through SIAK, the government generates NIK to all individuals in the population. More and more, birth registration became a “three-in-one” mechanism that involves SIAK generating an NIK for a newborn, SIAK registering the newborn on their family registry, and SIAK issuing a birth certificate.

### The role of the health sector in CRVS system

In Indonesia, birth registration requires a birth notification letter, commonly issued by a health facility or health workers assisting a delivery. Aside from one stipulation about the importance of the perinatal death records for health planning, the relevant laws on civil registration do not make any reference to the health sector. At the ministerial level, some ad-hoc agreements do define the role of the Ministry of Health (MoH) in birth registration. In 2011, MoHA with seven other ministries including MoH signed a memorandum of understanding (MoU) on achieving universal birth registration by 2015. This MoU was renewed in 2015 to achieve the national target on the birth certificate by 2019. The MoU assigns the MoH to mobilize health workers, especially birth attendants, to help the parent to register births and to raise public awareness about the importance of birth certificates. However, there is no technical guidance provided for health workers to perform these roles.

At the programmatic level, MoH does not use birth and death data produced by MoHA. They collect birth and death data through bottom-up reporting mechanism that starts from the midwives at village level all the way up to MoH at the national level. At each administration level (sub-district, district, province, and national), the records are aggregated. In 2010, MoHA and MoH entered into a joint agreement to generate comprehensive death statistics through data sharing. There should be an exchange of information regarding death events between the village apparatus and health personnel. In theory, compiled data will be reported to the District Office of Health (Dinas Kesehatan) which will share the data to Disdukcapil.

### Decentralization and healthcare

After the decentralization process in the early 2000s, the national government delegated most of the healthcare management to the district governments. Dinas Kesehatan at the district level is tasked to plan, fund, and deliver healthcare services, including managing the human resources at the sub-district community health center or *puskesmas* [27, 28].

Under this arrangement, one midwife coordinator is located at every *puskesmas* supervising at least one midwife in every village under the respective sub-district to provide basic healthcare [29]. The latest data from MoH shows that as of 2016 there were 163,541 midwives, of which 20,805 were working in remote villages [30]. Additionally, villages sometimes recruit members of the community as volunteers to assist midwives during routinely integrated health campaigns or *posyandu* [31]. In contrast, most civil registrars can only be found at district capital.

In addition to managing healthcare deliveries, MoH also manages the national health insurance (JKN) [28, 32]. JKN was launched in 2014, and it aims to provide financial support to all citizens in accessing quality healthcare including secondary and tertiary treatment. Before JKN, the health security was characterized by fragmentation with national and local government’s schemes run in parallel and rarely coordinated [33]. Their programs were predominantly residual and the offering of free primary and in- and outpatient treatments was only applied for those classified as poor [34].

Indonesia targets to register 95% of the population into JKN by 2019. The central government covers the premiums for low-income households under a scheme called PBI-JKN to ensure accessibility. Also, existing local health insurance schemes (*Jaminan Kesehatan Daerah*, shortened as “Jamkesda”) are incorporated into JKN [32]. Pangkep was one of the early districts to start integrating their local health insurance into JKN. This study, therefore, chooses to explore Pangkep in greater depth as administrative registration is immanent to support the expansion and integration of Jamkesda into JKN. This process is helpful to create awareness among healthcare forces about the importance of legal identity documents, which in turn can lead to birth registration potentials.

## Method

### Site selection

Pangkep district was selected purposively due to the ongoing integration of its local health insurance into JKN during the data collection of the original study. At the same time, Pangkep also has a relatively low birth registration rate during data collection. Analysis of Susenas 2015 shows that almost 28% of the children aged 0–17 years old couldn’t show their birth certificate, of which 77% of them was coming from the poorer households (Central Statistics Agency: National socioeconomic survey 2015, unpublished). Pangkep also represents geographical challenges as it consists of main capital island and hundreds of small islands spread around the main island. One sub-district was selected from Pangkep, and it consists of six villages that stretch across more than 20 small islands. The nearest island to the district capital, where Disdukcapil is located, is a 20-min commute by boat, while the furthest is about two hours away. The subdistrict was selected based on a set of criteria developed by KOMPAK, a partnership between Australian and Indonesian governments on governance, and the Government of Indonesia that consists of low scores on the national composite poverty index, strong buy-in from local leaders, and geographic variation.

### Participants

Semi-structured interviews were conducted with a purposive sample of health workers at the province, district, sub-district, and village level. At the provincial and district level, the team conducted a buy-in meeting facilitated by local planning and development agency (BAPPEDA) where the heads of Dinas Kesehatan were invited. The researchers identified the informants through conversation with the head of Dinas Kesehatan, based on the relevance of CRVS to their roles and responsibilities. At sub-district and village level, researchers applied snowball sampling and referred to the recommendation from community members in selecting informants.

### Data collection

Two trained researchers conducted key informant interviews with each participant using a semi-structured interview guideline (please see Additional file 1: Semi-structured Interview Guide for Health Sector). The participants were selected purposively based on their position on each administrative level: province, district, sub-district, and village. Prior to data collection, researchers attended two days training to familiarize with the instrument and practice the interview. All interviews were conducted face to face in Bahasa Indonesia. There was no prior relationship between participants and researchers and participation was sought without any help from higher health authority. Health workers at sub-district and village level were mostly sought from residents. Before the interview started, the researcher explained the purpose of the interview and sought consent. The researcher emphasized the confidentiality of the interview and the voluntariness principle of the study. Participants were told that they could skip, pause, or withdraw anytime without any consequence. While one researcher conducted the interview, the other audio-recorded the interview and took notes to compliment the audio transcript. There was no third party that presents in interviews except for one interview with village midwife where she was accompanied by her husband. There was also no refusal from participants, but some recommended participants were replaced by their coworker due to unmatched schedule. The interview mostly took place in the participants’ office or workplace and lasted from 30 to 90 min. There was no repeat interview, but there were several follow-up interviews for clarification or further information to reach saturation. We categorized the health workers into two levels: management which includes officials at the province and district level, and frontline which consist of health workers at sub-district and village level (see Table 1).

**Table 1:** List of Key Informants

Position	Government level	Number of participants		
		Male	Female	Total
Managerial	Province	3	1	4
	District	2	3	5
Frontliner	Sub-district	1	4	5
	Village	0	9	9
Total		6	17	23

### Analysis

Independent transcribers were hired to transcribe the audio files. To ensure confidentiality, the transcribers were asked to sign confidentiality agreement. Full transcription of all the audio was done to get comprehensive information from each respondent. Three researchers, all native speakers of Bahasa Indonesia, reviewed and cleaned the transcript before analysis. We depersonalized all the transcripts, the quotes, and removed additional information that could expose our participants prior to any publication. Thematic analysis approach informed the investigation of the data [35]. The analysis was conducted in Bahasa Indonesia involving an initial review of several selected transcripts to build preliminary codebook. These preliminary transcripts were selected based on the representation of each health administrative level and the richness of the information. The preliminary codes from selected transcripts were developed by reflecting on the research questions and interview guiding questions. All of the transcripts were subsequently examined to refine the codebook until no new code emerged. This process also includes merging, splitting, and renaming codes as more transcripts were analyzed. We applied the final codebook to the whole set of transcripts that were split between three researchers using Dedoose qualitative coding software. Each transcript was coded by two researchers to increase internal reliability and disagreements were addressed until we reached consensus.

The final codebook consists of 19 codes and then these codes were categorized into four main themes: (i) existing knowledge, value, and practice, (ii) supporting factors, (iii) potential challenges, and (iv) ways forward. Existing knowledge describes how respondent’s works related to CRVS including how they perceived the value of CRVS. Several codes related to this theme such as: perceived role in civil registration, recording and reporting, existing coordination. Supporting factors are the aspects that encourage respondents to be willing to involve in birth registration. Several codes associated with this theme such as “health benefit,” “data needed,” and “the use of civil registration”. Potential challenges describe the barriers and respondents’ concerns if they were to be involved in civil registration. Some codes related to this theme include “additional burden,” “lack of ownership,” and “internal challenges.” Ways forward refer to respondent’s notes/suggestions on how they might involve in CRVS in the future. The codes “potential role” and “recommendation” are associated with this theme. Apart from the major themes, this study did not discuss diverse cases and this paper, therefore, does not present any description of minor themes emerged from the data.

This study did not involve the informants in the analysis and had yet to report the results of the study back to the participants of the study. Preliminary findings of the primary study were presented to key stakeholders, both at the national and subnational level, where representations from Dinas Kesehatan from the study’s select districts attended and provided feedback during the discussion.

## Results

### Health workers’ understanding and perceived value of CRVS and birth registration

Most of the participants were familiar with legal identity documents such as birth certificates, ID card (KTP), and family card (KK). Frontline health workers were especially versant in birth registration and were aware of their responsibility to issue birth notification letter as one midwife put it “One of the requirements to obtain the *[birth]* certificate is a letter from the midwife, the birth notification letter.” They also voluntarily informed parents about the importance and the process of birth registration.

Many respondents said that these voluntary involvements were motivated by the health workers’ understanding about the impact of patients’ lack of proper identity documents on their ability to provide healthcare. One midwife at sub-district level, who has been working in that area for more than four years, said that midwives were often reprimanded by the hospital administrator if they brought patients (for referral services) without proper identity document.

*“But they didn’t process their KK. It would complicate things. If they become ill, that’s when we, midwives, have to play a role. Because we’re going to be the one who brings them to hospital but we’ll get reprimanded since the patient does not have any identity. That’s the problem.”*

Without their proper identity, the hospital would not be able to identify the patients’ eligibility for free health service and to register them accordingly. To register to JKN, individuals are required to provide an NIK that is displayed on identity documents such as IDs or KK. With a JKN, everyone from the low-income group who needs secondary or tertiary treatment can enjoy them for free upon a referral from *puskesmas*, except in an emergency where they can go directly to hospitals. Midwives who were posted at villages were often the ones who accompanied patients to access emergency service in the hospitals, and as such, they were often asked to be responsible for the patients’ administrative requirements even though it is part of the client’s personal responsibility. Therefore, for frontline workers, identity document is key in providing the optimum healthcare since the inability to register to JKN can be economically debilitating for their patients.

The ongoing consolidation of JKN in Pangkep has increased the urgency for health workers to ensure that all individuals in their area have the required identity documents to register. This awareness was translated into individual initiatives by frontline health workers to suggest parents to register their children’s births. Some of them also nudged parents to register their children to facilitate access to free health service, should their children need it in the future. For instance, the same respondent who experienced being reprimanded explained that she usually forewarned parents of a newborn about the importance of birth registration before or as soon as the birth.

*“When they were about to deliver, or just after the birth, we told them the information* *[on birth registration]*. *That the baby will need to be added to the KK. So, if the baby gets sick, well you know baby, under five years old is very prone to all kind of sickness. So, if they want to have things easy, if they do not want to pay* *[for potential medical services]*, *they have to process this identity.” (Midwife, sub-district level)*

In addition to ensuring people’s access to JKN, the need to increase in-facility birth delivery has also prompted one *puskesmas* to experiment as a mediator of birth registration for newborns. Promoting in-facility births is one of the MoH’s strategies to reduce maternal and infant mortality rate [36] and one *puskesmas* in Pangkep offered birth certificates as part of the incentive and could attest to its potency.

*“At that time, at the beginning of the year, the community was told that if you deliver your births here [at the puskesmas], we will process the birth certificate for you. Because well it is free of charge except for the transportation. And thanks be to God, there was an increase [in-facility births], but they could not keep financing [the registration]. It is too much, it is about 20km from there to [district capital] and they have not given any reimbursement for the transportation.” (Health personnel, district level)*

The absence of identity documents also impedes the integration of *Jamkesda* into JKN. KTP, KK, and birth certificates that display the NIK are needed to verify whether the individuals had already registered in JKN or not. In addition, KTP and KK are required to confirm their status as residents of Pangkep to secure their eligibility to a local subsidy for JKN. During data collection, Pangkep government just finished identifying beneficiaries which, according to provincial registry data shared by a key informant, consisted of 83,721 individuals. This figure was used as the maximum quota while a waiting list was developed to record eligible individuals who were excluded. The government planned to update the beneficiary list every semester by removing the deceased and the out-migrants and adding newborns, in-migrants, and people on the waiting list.

With that mechanism, maintaining an accurate record of population is increasingly important. Beyond birth registration, participants were concerned about the timeliness of their death records. They believe that accurate death reporting will prevent the government from misallocating JKN subsidy for the deceased. Lack of death registration, as one district health official expressed, could prevent the set subsidy quota from covering people on the waiting list.

*“It is crucial because we pay for it. That is why we need to update [the beneficiary list] every six months. If we can remove the dead people automatically, we can replace them with others. There are a lot of people on the waiting list. We’re just throwing money if the dead people are still listed. That is why an update is critical.”*

Participants were also aware that improving CRVS coverage including birth registration is necessary not only for JKN or increasing in-facility births. They see that the availability of birth data enables a more accurate measurement of child health indicators by calculating them against a closer-to-reality denominator.

### Health workers’ reluctance to register birth

Despite the appreciation and understanding of the role of functioning CRVS, participants differed in their opinion when it comes to institutionalizing health sector’s participation in the birth registration. Many health workers tend to disapprove of the proposal. For midwives, increasing involvement in birth registration might take away time and resources from their already burgeoning responsibilities.

*“Too much, it is cumbersome. I mean, let’s just direct [applicants] to village office. They have staff there, and they must help. If we are to take care of [birth registration], our burden is increasing.” (Midwife, subdistrict level)*

Some of the burdens that they carry are administrative. On top of the primary care workload, health workers were responsible for numerous routine reporting with many forms to fill. The majority of the documentation is also still paper-based.

*Facilitator: “Have you had any difficulty with all these reports?”*

*Participant: “It is confusing because there are too many reports.” (Midwife, village level)*

The majority of the participants considered civil administration as the responsibility of Disdukcapil and village official. For frontline workers, birth registration is the task of village apparatus, not health, although it is worth noting that some village offices that we visited were not equipped to assist with registration.

As to the managers, they were more concerned about being seen as encroaching on the authority of another sector.

*“We can help, but the power [to register] is within the Disdukcapil and Dinas Sosial [the social welfare agency] if it is for financing [JKN]. We are afraid that they think we have hidden interest. Our mandate is to provide health care. To register is other department’s [duty].” (Health personnel, district level).*

### Opportunities to involve health workers in birth registration

Nevertheless, we found that some participants, especially from the managerial level, were more open to the idea of engaging health workers in birth registration systematically. One district health official thought it is a good idea, noting that the health sector has a bigger number of personnel who work closely with the community.

*“We can help, of course. Health sector has staff even at the village level, the village midwives, in all villages. But we do not have the authority.”*

During data collection, all villages that were visited have at least one midwife and several health volunteers. In many villages, midwives also operate ancillary health clinics called *pustu* and *poskesdes*, often with the assistance of one or two nurses.

Participants also identified some tasks that can be streamlined into midwives’ and health volunteers’ routine work. The suggestions include identifying unregistered babies as well as providing information to parents on the importance and the procedure to get a birth certificate. One district health official recommended adding a new checklist column on birth certificate ownership in the midwife’s register to systemize the process.

*"We can expect volunteers to provide information. We can also add a checklist in their books, to identify whether the child already has a birth certificate or not. And this can be done either during pregnancy check-ups or when the child comes for weighing.”*

Another participant expressed that the role of health workers could be expanded to facilitate birth registration by referring the needs to Disdukcapil. As an incentive, using the child’s birth certificate as proof every midwife should provide alongside their report on birth delivery service was suggested.

Participants at management level advised several precautions before any engagement with health workers on birth registration. First, there should be a precise regulation that specifies the nature of the collaboration between Disdukcapil and health workers and their respective roles. “Any collaboration”, one district health official mentioned, “cannot work without regulation from top to down.”

MoU between participating sectors at the local level as an alternative to top-down regulation is recommended by one district health official while another one mentioned forming a working group at the frontline level would facilitate the collaboration at the implementation level.

*“[Midwives’ involvement in birth registration] is something that we need to think and to develop. The management of this will be important. It depends on a working group. The authority is currently with the civil registration office. So, we need to discuss whether [the registration office] just want to receive application from family or, they can receive an application for birth certificate right after births [from health workers].”*

In any case, participants emphasized that regulation should always be followed by efforts to increase the capacity and supports for health workers.

*“Well, if it’s only regulation without facilities, no knowledge, no support, well what you would make of that…” (Health official, district level).*

One participant also added that any increased involvement of health workers in birth registration must be accompanied with increased outreach from Disdukcapil. One example put forward by one district health official was for Disdukcapil to dedicate one person to liaise with health workers at sub-district level. It was described as follows:

*“Perhaps we can make a MoU, and it’s recommendable that there’s also an increased outreach from Disdukcapil because if it’s all puskesmas, that’s too much for them to dedicate one staff just for this. All staff there take up additional roles, so frankly, it’s a bit too overwhelming. So I think puskesmas can help, but there’s an officer from Disdukcapil to come handling it directly to puskesmas.”*

## Discussions

In Indonesia, the decentralization puts the onus of primary services on local government. A few districts are experimenting with various efforts to engage the health sector in birth registration. In Aceh Besar district, there is a collaboration between the health and civil registration sector, where healthcare personnel helps parents to prepare birth certificate and KK applications for their newborns [37]. In the city of Surakarta, under an MoU between hospitals and clinics with the civil registration office, healthcare staff not only provide information to patients but also input birth information into a modified version of SIAK for civil registration office to follow up with verification and certification [5]. The government of Jakarta recently launched a three-in-one service where all newborns will be given a birth certificate and registered in JKN before they leave certain hospitals [38].

In contrast to examples from other districts, the health workers in Pangkep have already done some of these functions on a discretionary basis without top-down direction. These engagements might partly be attributed to the feeling of responsibility to the community that is prevalent among midwives who chose to work in remote areas [39]. Aside from that, frontlines also wanted to ensure they did not face issues related to health insurance due to patients’ lack of legal identity documents. These voluntary engagements, however, hardly contribute to increasing the birth registration coverage over time, thereby limiting its indirect significance on the production of vital statistics.

**Fig. 1:**
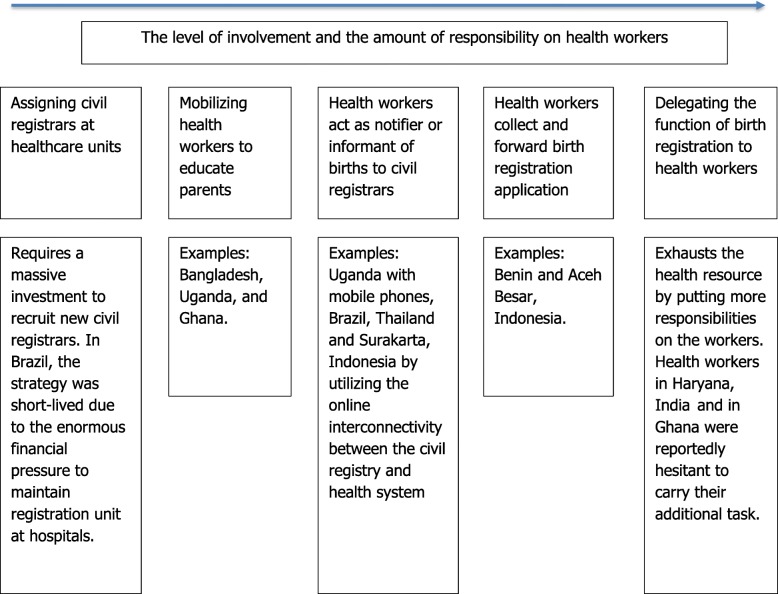
The type of health sector’s engagement in birth registration [5, 40, 20, 22, 23, 38, 41–43]

The type of engagement will determine the amount of additional responsibilities placed on frontline health workers. In general, there are three strategies to involve the health sector in birth registration (see Fig. 1). At one end, health workers are barely involved and the government posts civil registrars at health service units, while at the other end, health workers take over the function to register births. In between these two extremes, health workers can be engaged to facilitate birth registration in several ways.

Our findings show that there was an agreement that the role of health workers in facilitating birth registration was to dispense information and to raise awareness, not to undertake the registration itself. There was a common sentiment that adding a birth registration function on health workers would be a significant burden to their already huge workload and infringing on another sector’s mandate. This finding corresponds to their suggestions that any further involvement of health workers must always be followed with increased outreach from Disdukcapil. In addition, *puskesmas* that operate at sub-district level can act as a node for coordination since all the village midwives attend a meeting at *puskesmas* regularly. This arrangement will also reduce the added burden placed on the health sector and its workers.

**Fig. 2:**
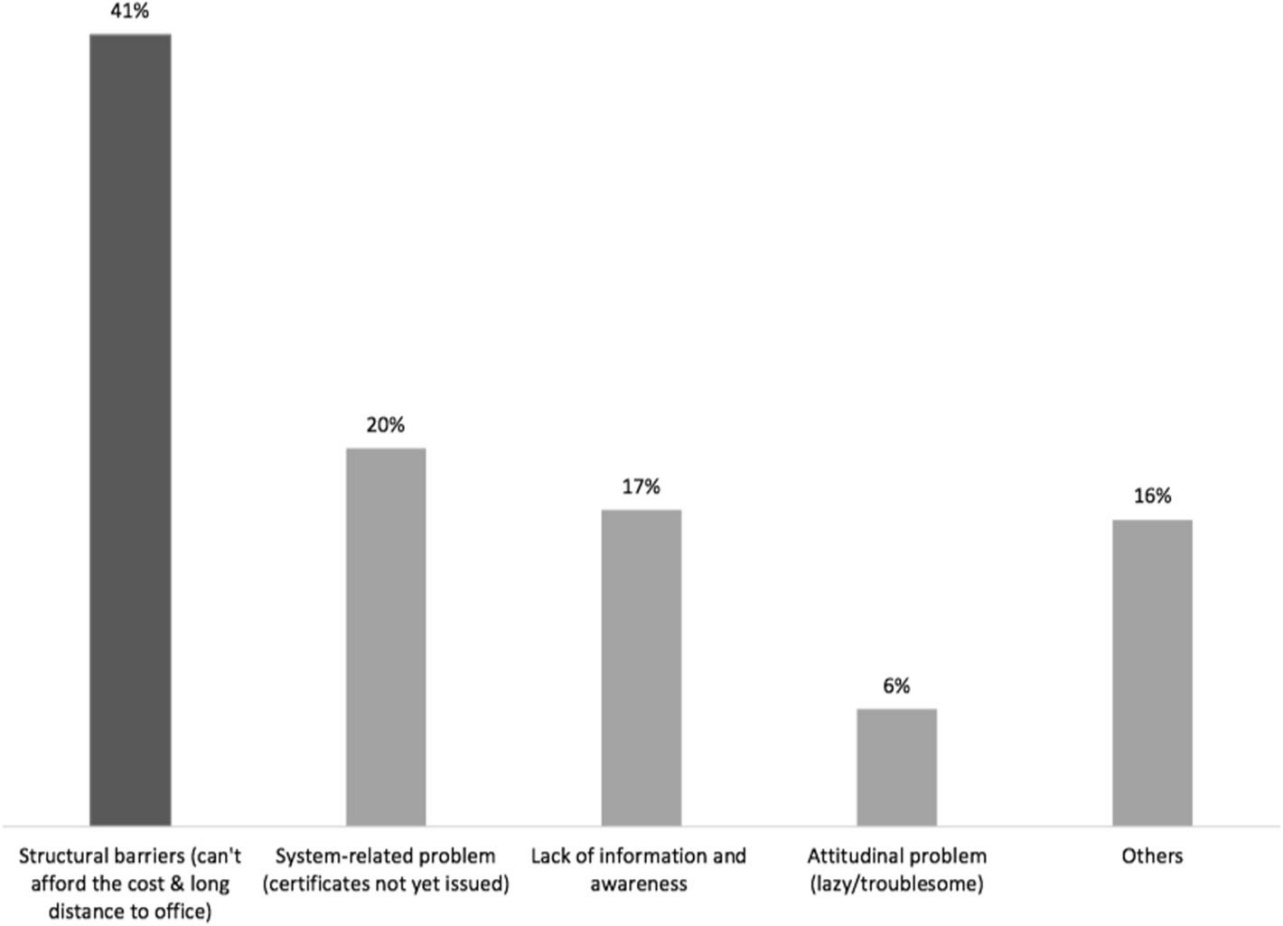
Reasons for not having birth certificate (source Susenas 2016)

The Susenas data of 2016 confirms such needs (see Fig. 2). The main barrier to birth registration is the cost of registration (34%) while 7% cited distance as the main hurdle. Since birth registration is free of charge, most of this cost is associated with transportation money, the opportunity cost of spending time on the registration, and other related expenses [6, 24, 44]. While the second main barrier is related to supply-side (20%), the next main barrier is closely related to information and awareness deficit (21%). If village midwives and nurses were mobilized to assist with birth registration by relaying information and brokering parents and civil registrars, it could potentially improve the coverage.

It is crucial to provide a legal basis for the systematic involvement of health workers in birth registration [21]. This legal basis should detail their new mandates, and how it will complement rather than replace the responsibilities of the civil registration office. It is also equally important to ensure that this mandate is recognized at every level of government. In a decentralized system, regulation and agreement between ministries do not automatically command desirable changes at implementation level as local counterpart of the ministries are mainly accountable to the head of district [45]. For instance, we found that none of the participants was aware of the MoU of seven ministries to achieve universal birth registration and the joint regulation between MoH and MoHA on mortality data sharing. Issuing similar agreement and forming a working group at the local level are some ways of ensuring enforceability although this might mean relying heavily on the political appetite of local leaders to implement certain reforms [46].

Effective involvement of the health sector in birth registration requires investment in increasing health workers’ knowledge and skills, not only in birth registration but in CRVS more broadly, focusing on their roles in the system. Routine training is one standard way to achieve this as is shown in Bangladesh [21]. Periodic supervision, as it is already in practice in Indonesia, can be done as part of the regular cross-sectoral coordination meeting held by *puskesmas* once every few months.

Finally, there is a question of incentivizing health workers’ involvement in aiding birth registration. In Brazil, monetary reward for health units per child registered was part of the package to incentivize health workers [21]. However, this comes with a caveat; that it might risk taking away human resources from delivering the primary task of health care.

## Conclusion

Many country analyses have demonstrated the effectiveness of the various strategies to involve the health sector in birth registration. This study complements the existing scholarship by providing an empirical insight on how health workers, especially from a geographically challenged area, value birth registration, how they have been involved, and how they perceive their role more systematically in birth registration.

The Indonesian health workers at frontline and management levels who participated in the study do appreciate a functioning birth registration and even broader CRVS system and understand their significance in their line of duties, both for service delivery and planning purposes. They were also aware of their comparative advantages in outreach to assist birth registration. However, the health workers at the frontline level were not necessarily sanguine about being engaged systematically in birth registration although some of them occasionally already facilitated birth registration.

While this study appreciates the potential of institutionalizing the health sector’s engagement to achieve universal birth registration and, even further, health insurance, any attempt toward it needs to consider some conditionalities. First, the risk of adding the workload of health workers. Second, even by regulating specific roles health workers should play in birth registration, balancing this additional task with rewards and support should be carefully calculated as not to create a moral hazard that will pull health workers away from their healthcare tasks.

Although this study is limited to one district, this paper argues that it offers preliminary insights that are representative to the health sector’s perspective of birth registration at a local level in Indonesia as all districts in Indonesia follow similar structure of the health service and civil registration system. However, the existing body of knowledge will benefit from replications of this study in other districts as a way to document variation of localities. Since this study focused on the health workers and managers’ point of view at the district level, future studies are needed to further understand the perspectives of the civil registration sector and the administrative and political challenges at the national level that may support or prevent an effective collaboration between the health and civil registration sector. Furthermore, there is a need to study the efficacy of the health sector’s engagement in birth registration as well as in other vital registrations.

## Supplementary information

**Additional file 1.** Semi-structured interview guide for health sector. This document contains the guiding questions that researcher referred to when they conducted interviews with participants from health sector.

## Data Availability

The authors are willing to share the interview transcripts upon considering individual requests to PUSKAPA (puskapa@puskapa.org) or one of the authors. We cannot make transcripts available open access given the sensitive nature of the information shared and the possibility to easily identify respondents due to the small sample size and heterogeneity of respondents.

## Abbreviations

BAPPEDA: Local planning and development agency
CRVS: Civil registration and vital statistics
Disdukcapil: Dinas Kependudukan dan Pencatatan Sipil (district office of population and civil registration)
Jamkesda: Jaminan Kesehatan Daerah (local health insurance)
JKN: Jaminan Kesehatan Nasional (national health insurance)
KK: Kartu Keluarga (family card)
KTP: Kartu Tanda Penduduk (individual identity card)
MoH: Ministry of Health
MoHA: Ministry of Home Affairs
MoU: Memorandum of understanding
NIK: Nomor Induk Kependudukan (unique identity number)
PBI-JKN: Penerima Bantuan Iuran Jaminan Kesehatan Nasional (national health insurance premium subsidy scheme)
Poskesdes: Pos kesehatan desa (village health post)
Posyandu: Pos pelayanan terpadu (integrated health service post)
Puskesmas: Pusat kesehatan masyarakat (community health center)
Pustu: Puskesmas pembantu (community health center aide)
SDGs: Sustainable Development Goals
SIAK: Sistem Informasi Administrasi Kependudukan (population administration information system)
Susenas: Survei sosial ekonomi nasional (national annual socio-economic survey)
WHO: World Health Organization
