# Supplementary material for: Strategic yet delicate: the dilemma of involving health workers in facilitating birth registration in Indonesia
Source: BMC Health Serv Res. 2019 Nov 26;19:889. doi: 10.1186/s12913-019-4594-z (PMC6880581; doi:10.1186/s12913-019-4594-z)
Supplement: Supplementary file 1 — Additional file 1. Semi-structured interview guide for health sector. This document contains the guiding questions that researcher referred to when they conducted interviews with participants from health sector. [file 12913_2019_4594_MOESM1_ESM.docx]

***Strategic yet Delicate: The Dilemma of Involving Health Workers in Facilitating Birth Registration in Indonesia***

**Semi-structured Interview Guide for Health Sector**

**Introduction:**

You will have already discussed the project with most of your informants prior to your meeting, but give a brief introduction to the research. Do not mention the importance of birth certificates, or any other value judgments about civil registration. You should be neutral about these so as not to influence the informant. Our purpose here is to understand the relationship between civil registration and vital statistics and the provision of other basic services. Remind them that their responses will be confidential but that you want to record for research purposes. Ask if this is okay, and explain that the interview should take no longer than an hour.

- If the informant agrees to be recorded, turn on the recorder and say the date, your name, and the title of the person you are interviewing along with their organization.
- If the informant does not agree to be recorded, explain that you will then simply take notes – and record this refusal in your notes together with the date, your name, and the title of the person you are interviewing along with their organization.

**Reminders:**

- Ask about death and birth separately and make sure responses clearly pertain to one or the other
- Add new questions to this document that you’ve thought of during the interview
- Mark questions that you have not had a chance to ask and look for opportunities in your next interview to ask them

**Opening questions**

- Could you please describe what you do on a daily basis for work, and how this intersects with civil registration?
  - Do you feel that registering births/deaths is part of your job responsibilities? (Why? Why not?)
  - If no to the above: have you ever facilitated birth/death registration for someone in this community? (How did you facilitate this? Why? Did you receive support for this? Who did you work with to accomplish this? Why haven’t you facilitated registration? Why did you stop facilitating services?)
- How is this related to larger systems of registration? (Use general questions below to probe)

Move on to general questions below.

Then move on to topic-specific questions depending on key informant and ask all mandatory questions.

| **Topic** | **Question** |
| --- | --- |
| General | - What opportunities can you identify in your job for informing parents about the value and process of attaining birth certificates for their children? - What are ways that you might be able to use CRVS data to improve your services? - What are ways that your services might be adapted to improve birth/death registration? What kind of support would you need for this from higher levels of government? - What opportunities can you think of to work with other sectors on registration activities? What kinds of challenges would there be in collaborating with these other organizations? - What are birth certificates required for in this community? Do you think people get birth certificates to meet these requirements? Are there any ways to get around these requirements? - How important are death certificates in this community. Do you need death certificates for anything? Are burial permits required? Does anyone actually get death certificates? How about burial permits? - What would you say is the relationship between birth registration and disability in this community? - When would you say most mothers try to register their children’s birth? Are there any naming practices that delay birth registration? Are there any other cultural practices that may interfere with the registration process? Are there any cultural practices that may present opportunities for registering births? - When someone dies here, are they usually buried within 24 hours? Does this interfere with death registration? What are the processes for burying bodies in this community? Does anyone used these as opportunities to prompt families to register deaths? If someone wanted to use verbal autopsy here, how long after the death would it be appropriate to speak with the family of the deceased? |
| Health | - How often do you record births/deaths as part of your job? Where do you receive these data from? Where do you report these data to? Have you used these data for anything? How have you used these data in your job? - Do you keep a cohort book here? How long do you keep your cohort book? Do you record children’s NIKs in your cohort book, or any other information related to birth registration? - Do you ever work with the police department for accidents and crimes? Do you share information on deaths and causes of death? How do you share such information? Who shares with whom? Why don’t you share such information? Do you think it would be possible to share more information? Has the police ever asked for more information that you haven't been able to provide? What sorts of challenges have you faced in working with the police? As far as you know, does the police keep data on children born in prisons? What about neglected babies in the community? - How often does this community have conduct Posyandu? What data are recorded during Posyandu? Does this include information on births and deaths? Are these opportunities for registering children’s births and people’s deaths? Who is involved in Posyandu? |
|  | Maternal, Child, & Reproductive Health |
|  | - When a mother delivers a newborn, does she receive any information on birth registration? Who provides this information and how? Are there any mother and child classes? Is the mother prompted to register her child? Can she do so in this facility? Is there anyone at this facility who could facilitate this registration? - Do women in this community typically use antenatal care? - Who provides antenatal care? Does antenatal care involve disseminating information on birth registration? - Are buku KIA distributed to mothers? Where does this supply come from? Does it ever run out? How frequently? Do you have to pay anything for them? Does the mother have to pay anything for them? Does anyone explain the contents of the buku KIA to mothers? [If available, check the buku KIA to see that there is currently a stock and to check that there are statement letters in the buku KIA]. Do you use the statement letters in the buku KIA or are there other statement letters? If they don’t use the buku KIA letters, do parents have to pay for the other statement letters? - How commonly would you say people here use traditional birth attendants? What kind of relationship do these TBAs have with the health sector? Do TBAs ever receive training from health workers? If so, could they be used to have TBAs help register births/deaths? - How do children in this community receive vaccinations? Who is responsible? Can these campaigns be used to register children’s births? |
|  | Insurance |
|  | - When a mother has a child, how does she provide insurance for her child? How much time is she given to take care of this? Does the mother receive any help with this? If so, from whom? In your birth delivery packets here is there information about birth certificates? - If most people don’t have death certificates, how do you close people’s health insurance accounts? How do you settle pension claims or inheritance claims? |
|  | Death |
|  | - What happens if someone dies in this community? What are the procedures for processing this death? Who is involved? - If a child dies during delivery, how is that recorded? Who is responsible for recording it? Who pays for recording it? Where does this information go? How is it used? - If a mother dies during delivery, how is that recorded? Who is responsible for recording it? Who pays for recording it? Where does this information go? How is it used? - Has anyone here ever been trained to use International Causes of Death Coding or verbal autopsy? |
